# Supplementary material for: Hydrogen sulfide aggravates neutrophil infiltration, vascular remodeling and elastase-induced abdominal aortic aneurysm in male mice
Source: Commun Med (Lond). 2025 Jul 1;5:267. doi: 10.1038/s43856-025-00978-5 (PMC12217252; doi:10.1038/s43856-025-00978-5)
Supplement: Supplementary file 2 — Supplementary information [file 43856_2025_978_MOESM2_ESM.pdf]

Supplementary information

Supplementary Table 1: Primers

Gene	Forward	Reverse
Mouse Cth-P1_Fw	AGCATGCTGAGGAATTTGTGC	
Mouse Cth-P4_Rv		AGTCTGGGGTTGGAGGAAAAA
Mouse Cth-P2-LacZ_Fw	TTCAACATCAGCCGCTACAG	
Mouse Cth	TTGGATCGAAACACCCACAAA	AGCCGACTATTGAGGTCATCA
Mouse Cbs	GGGACAAGGATCGAGTCTGGA	AGCACTGTGTGATAATGTGGG
Mouse Mpst	GGCCACCACTCTGTGTCATT	GGAGCTGATTGGCAGGTTCT

Supplementary Figures

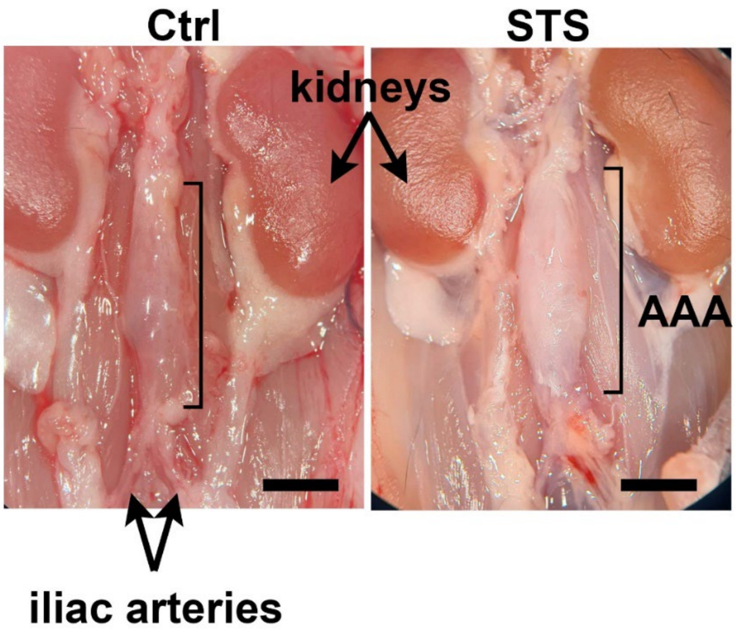

Supplementary Figure 1. STS increases AAA size in a mouse model of topical application of elastase. Representative photograph of sub-renal AAA 14 days pots-topical application of elastase in WT male mice treated with BAPN, and treated or not (Ctrl) with 4gr/L STS (STS). Scale bar = 2 mm

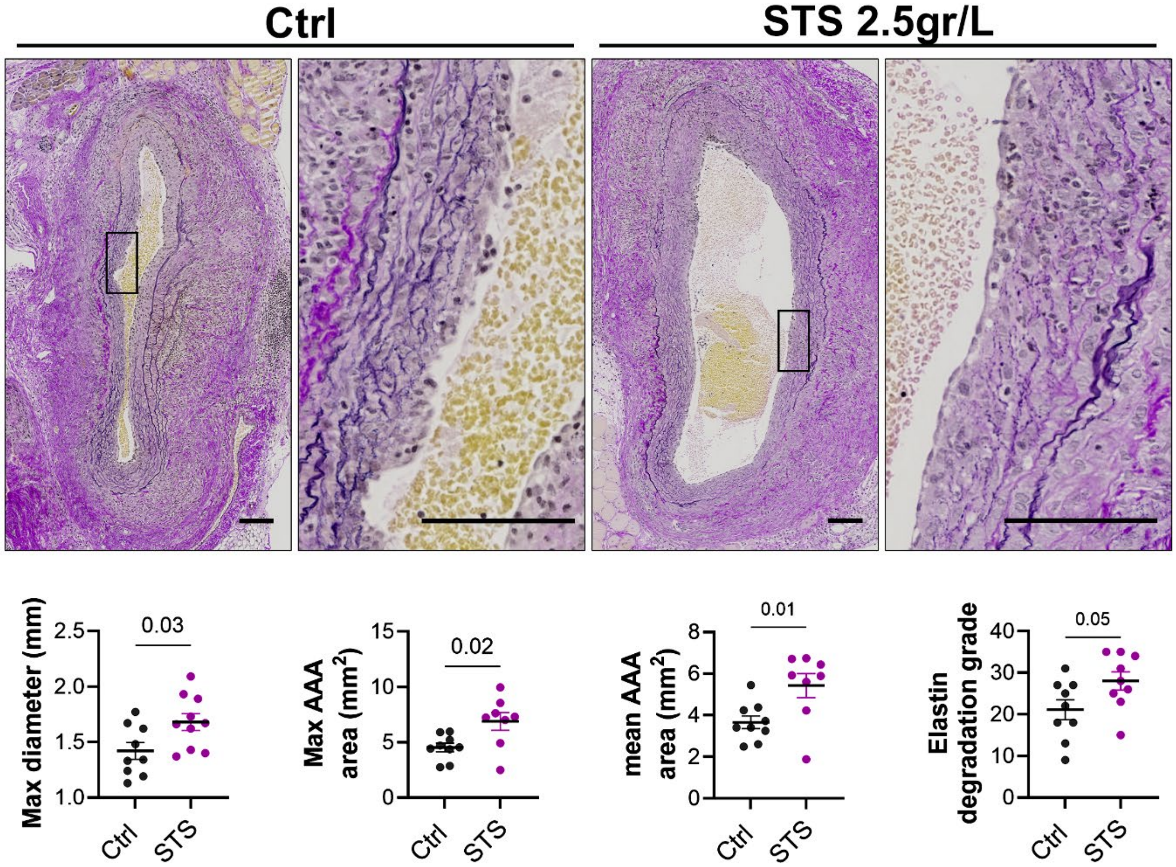

Supplementary Figure 2. STS increases AAA size in a mouse model of topical application of elastase.

Representative Verhoeff–van Gieson Elastic Lamina (VGEL) staining (*upper panels*) and quantitative assessment (*lower panels*) of max diameter, max and mean aorta lumen area, and elastin degradation grade in sub-renal mouse aorta in WT mice with topical elastase application, treated or not (Ctrl) with 2.5 g/L sodium thiosulfate STS. Data are mean±SEM of 9 Ctrl and 10 STS-treated animals. Scale bar = 100 µm. P values determined by bilateral unpaired t-test.

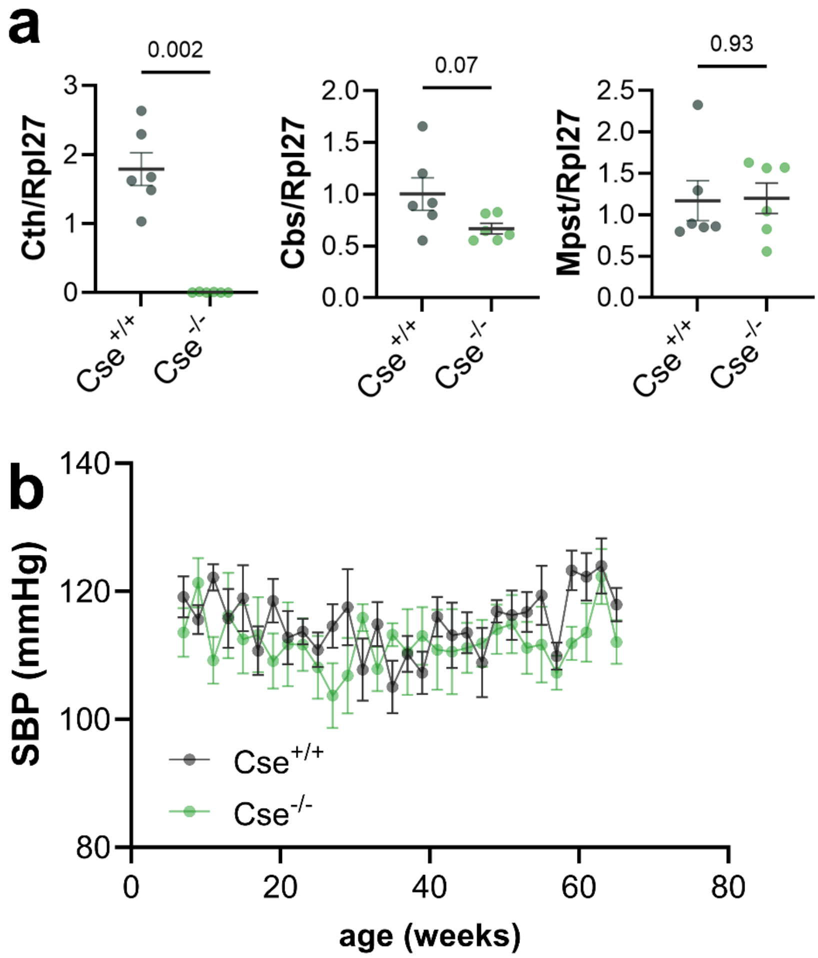

Supplementary Figure 3. Constitutive Cse knock-out mice ($Cse^{-/-}$) remain normotensive with age.

a) qPCR analysis of *mouse cystathionine gamma-lyase* (*Cth)*, *cystathionine beta-synthase* (*Cbs)* and 3-*mercaptopyruvate sulfurtransferase* (*Mpst)* in native aortas in $Cse^{-/-}$ and WT ($Cse^{+/+}$) male littermates. Data are mean±SEM of 6 mice per group. P value as determined by Kolmogorov-Smirnov test for *Cth* expression. No significant difference as determined by bilateral unpaired t-test for normally distributed *Cbs* and *Mpst* expression. **b**) Systolic Blood pressure (SBP) in $Cse^{-/-}$ and $Cse^{+/+}$ male littermates. Data are mean±SEM of 6 animals per group. No significant difference as determined by matched (repeated measures) mixed-effects model (REML).

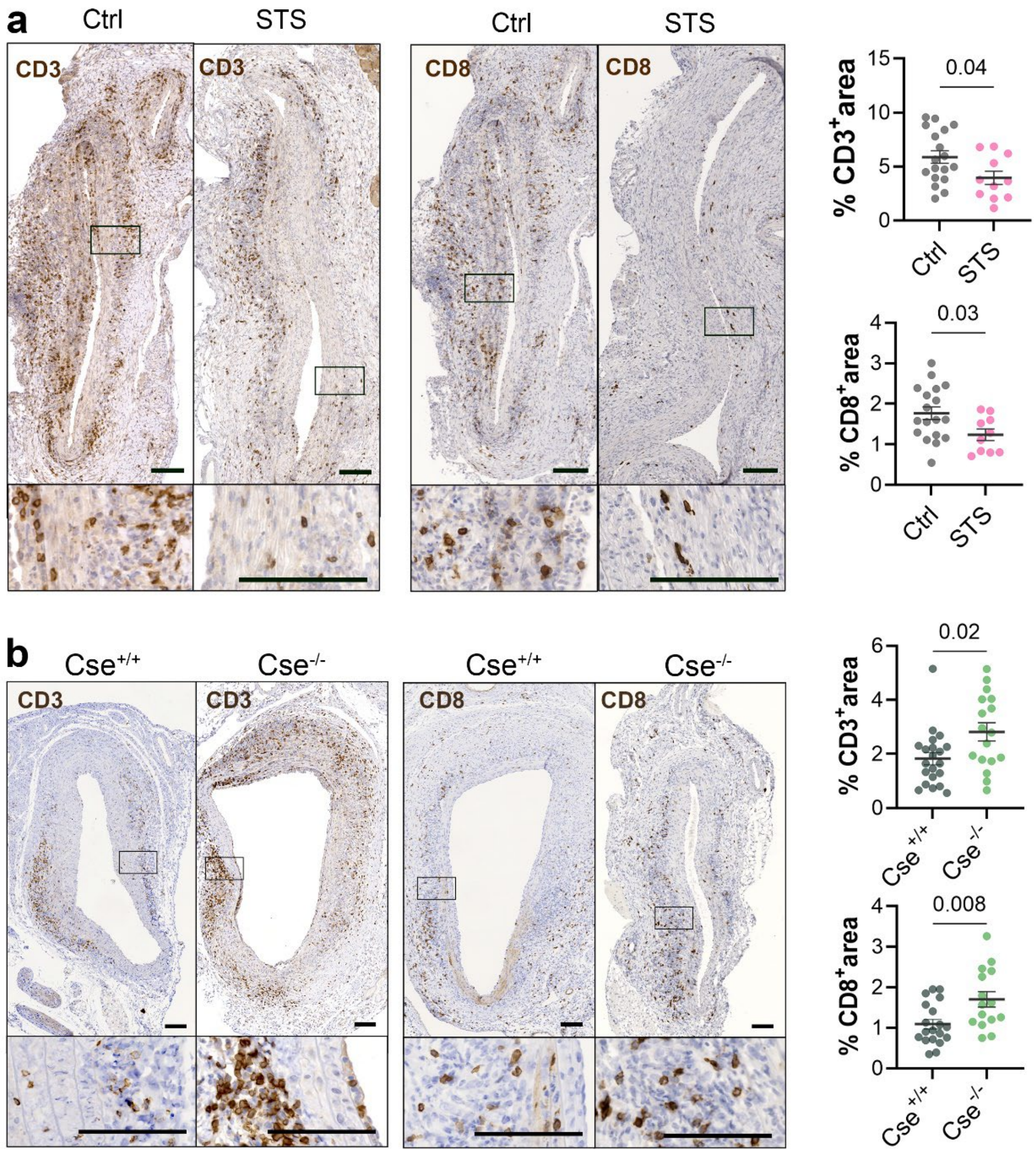

Supplementary Figure 4. H_2S decreases T-cells infiltration in the aortic wall.

a) Representative CD3 and CD8 immunostaining (*left panels)* and quantitative assessment (*right panels)* of t-cell infiltration in sub-renal mouse aorta in wild type male mice 14 days post topical elastase application, treated or not (control) with 4g/L sodium thiosulfate (STS) and β-aminopropionitrile (BAPN). **b**) Representative CD3 and CD8 immunostaining (*left panels)* and quantitative assessment (*right panels)* of t-cell infiltration in sub-renal mouse aorta in $Cse^{+/+}$ and $Cse^{-/-}$ male mice 14 days post topical elastase application and trested with BAPN. **a-b**) Data are mean±SEM of 18 Ctrl, 18 STS, 21 $Cse^{+/+}$ and 17 $Cse^{-/-}$ mice. Scale bar = 80 µm. P values determined by bilateral unpaired t-test.

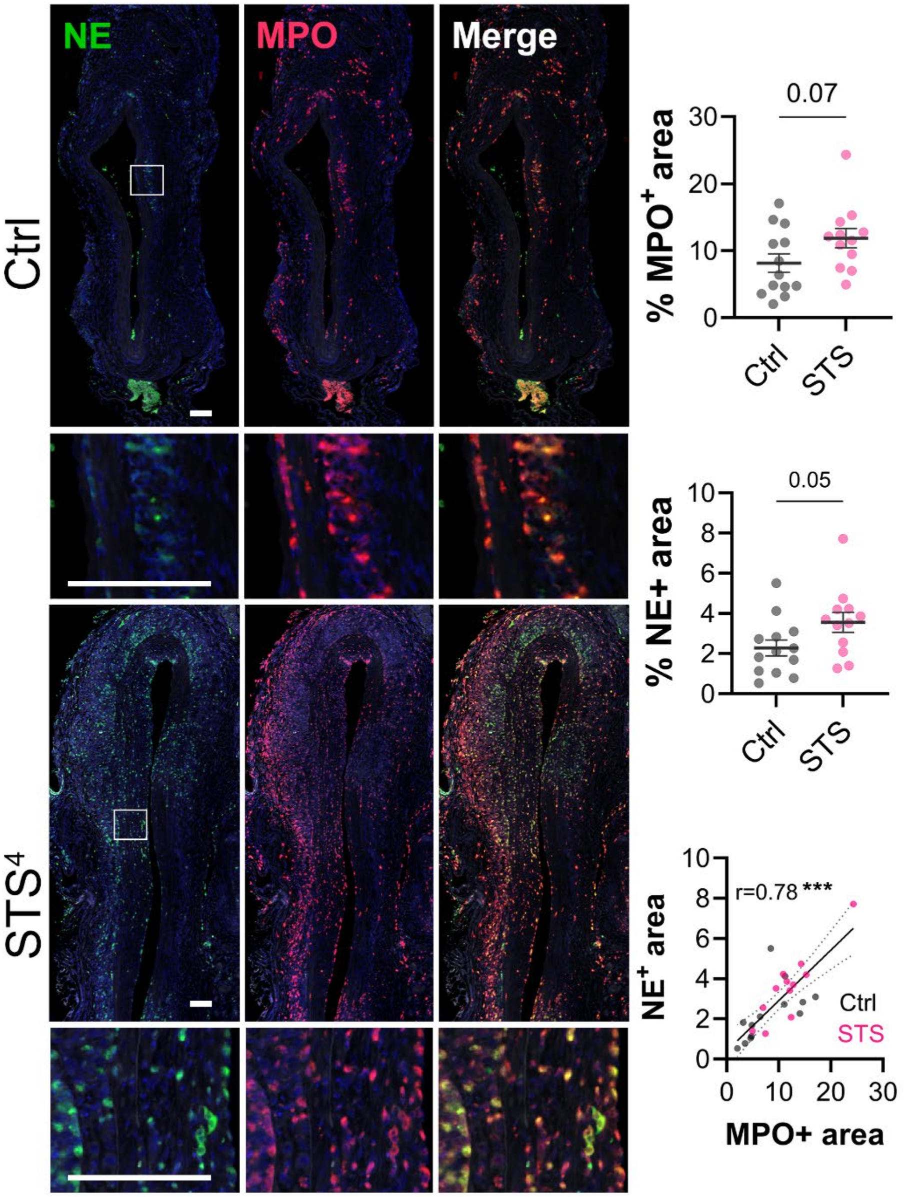

Supplementary Figure 5. 4g/L STS promotes infiltration of neutrophils.

Left Panels: Representative Myeloperoxidase (MPO) and neutrophil elastase (NE) co-immunostaining in sub-renal mouse aortas in male mice 14 days post topical elastase application and treated with β-aminopropionitrile (BAPN), treated or not (control) with 4g/L sodium thiosulfate (STS^4). Scale bars =80 µm. *Right Panels:* Quantitative assessment and correlation analyses of MPO and NE positive area. Data are mean±SEM of 13 Ctrl and 10 STS-treated mice. P values determined by bilateral unpaired t-test and Pearson correlation analysis (***p<0.001).

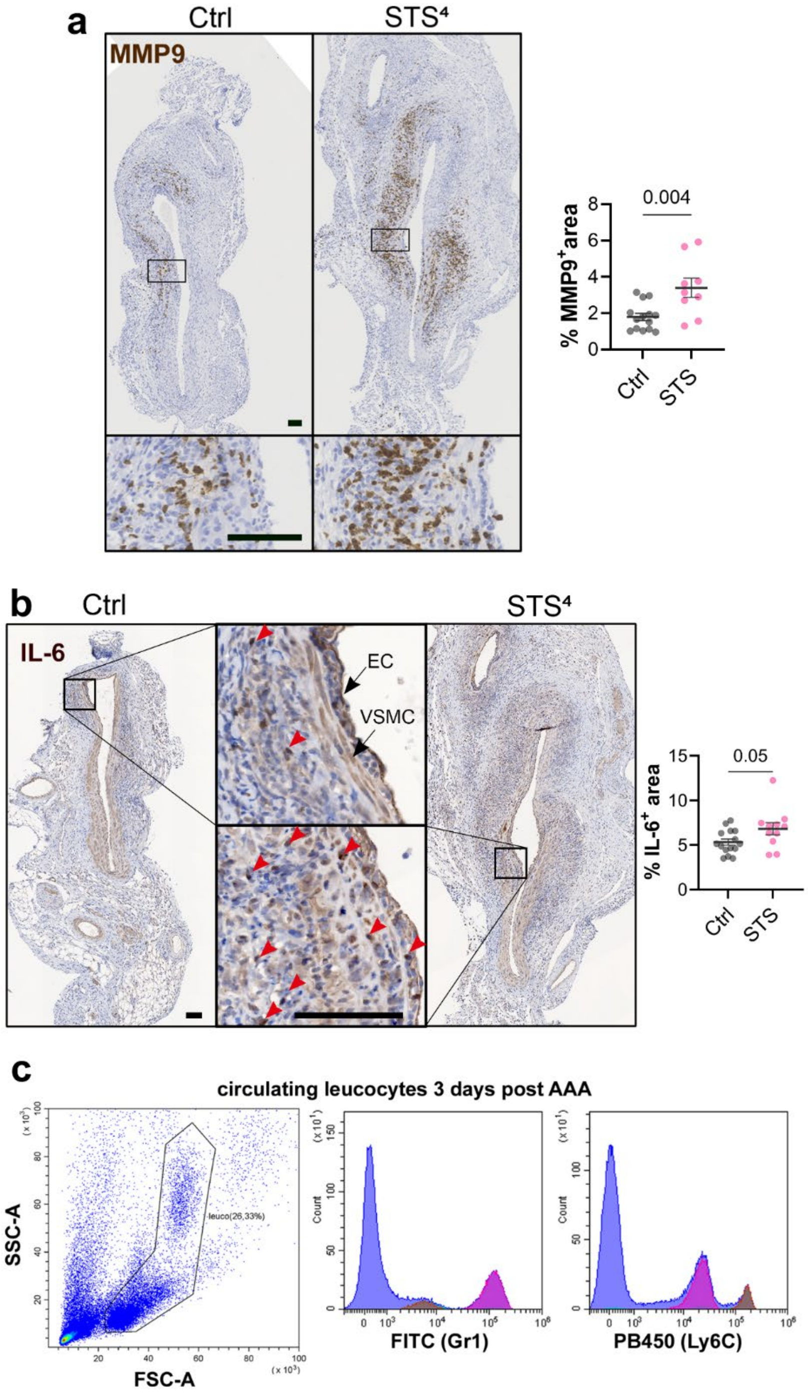

Supplementary Figure 6. 4g/L STS promotes infiltration of MMP9$^+$ neutrophil.

a-b*) Left Panels:* Representative Matrix metalloproteinase-9 (MMP9; **a**) and interleukin-6 (IL-6; **b**) immunostaining in sub-renal mouse aortas in wild type male mice 14 days post topical elastase application presence of β-aminopropionitrile (BAPN), treated or not (control) with 4g/L sodium thiosulfate (STS^4). Scale bars =80 µm. Red arrowheads highlight intramural IL-6$^+$ cells. *Right Panels:* Quantitative assessment of MMP9 (**a**) and IL-6 (**b**) immunostaining. Data are mean±SEM of 14 Ctrl and 10 STS mice per group. P values determined by bilateral unpaired t-test. **c**) Representative gating strategy and Ly6c and Gr1 staining on peripheral blood leucocytes 3 days post-surgery in wild type male mice. 10^5 events were recorded in the leucocytes (leuco) gate.

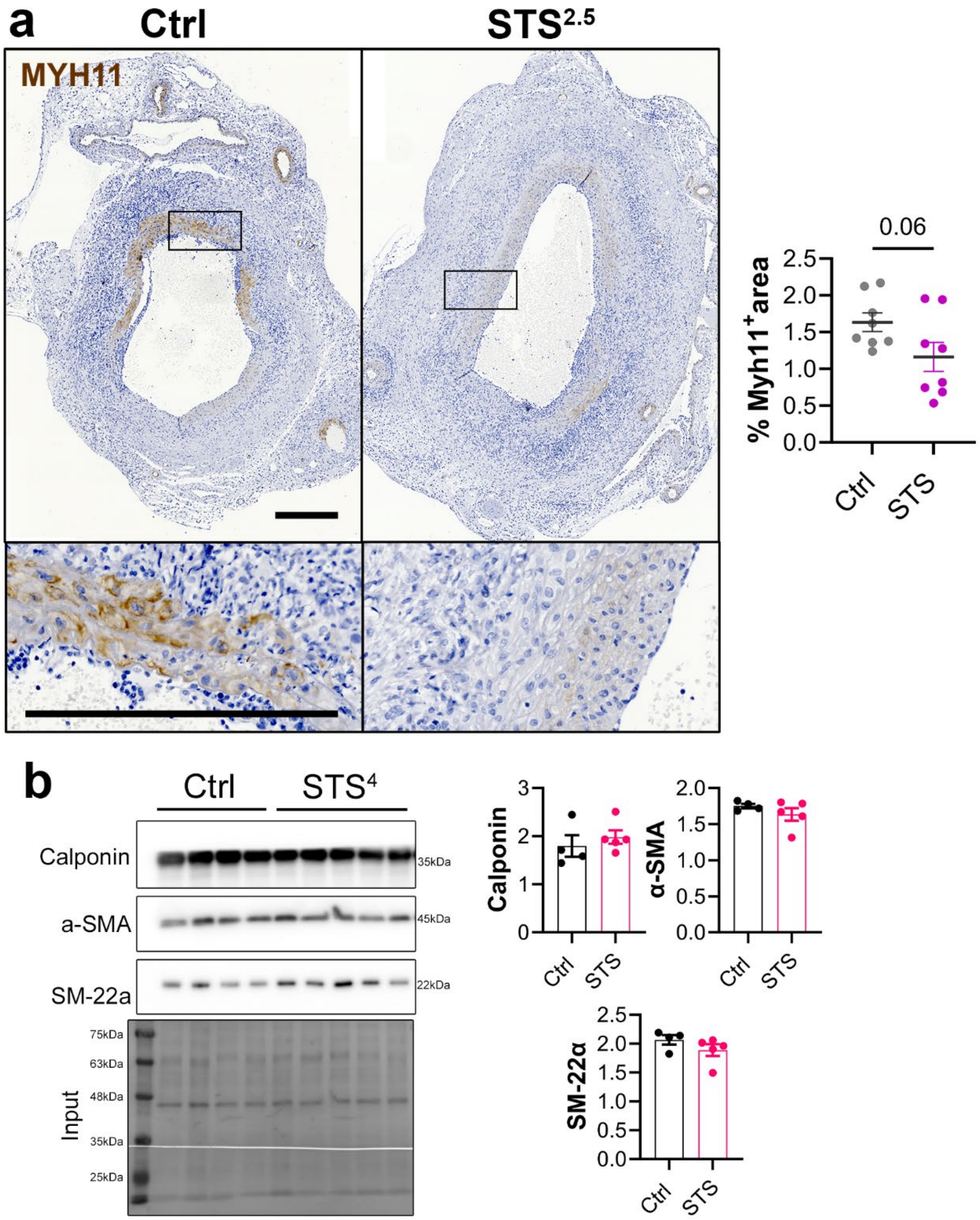

Supplementary Figure 7. STS does not impact VSMC phenotype but reduces MYH11⁺ VSMC coverage

a) *Left Panel:* Representative Myosin-11 (MYH11) immunostaining in sub-renal mouse aortas in wild type male mice 14 days post topical elastase application, treated or not (control) with 2.5g/L sodium thiosulfate ($STS^{2.5}$). Scale bar =200 µm. *Right Panel:* Quantitative assessment of MYH11 immunostaining in 8 animals per group. Data are mean±SEM. P=0.06 as determined by bilateral unpaired t-test. **b**) Western blot analysis in native aorta from mice treated or not (Ctrl) for 1 week with 4g/L STS. Data are mean±SEM of 4 mice per group. No significant difference as determined by bilateral unpaired t-test.

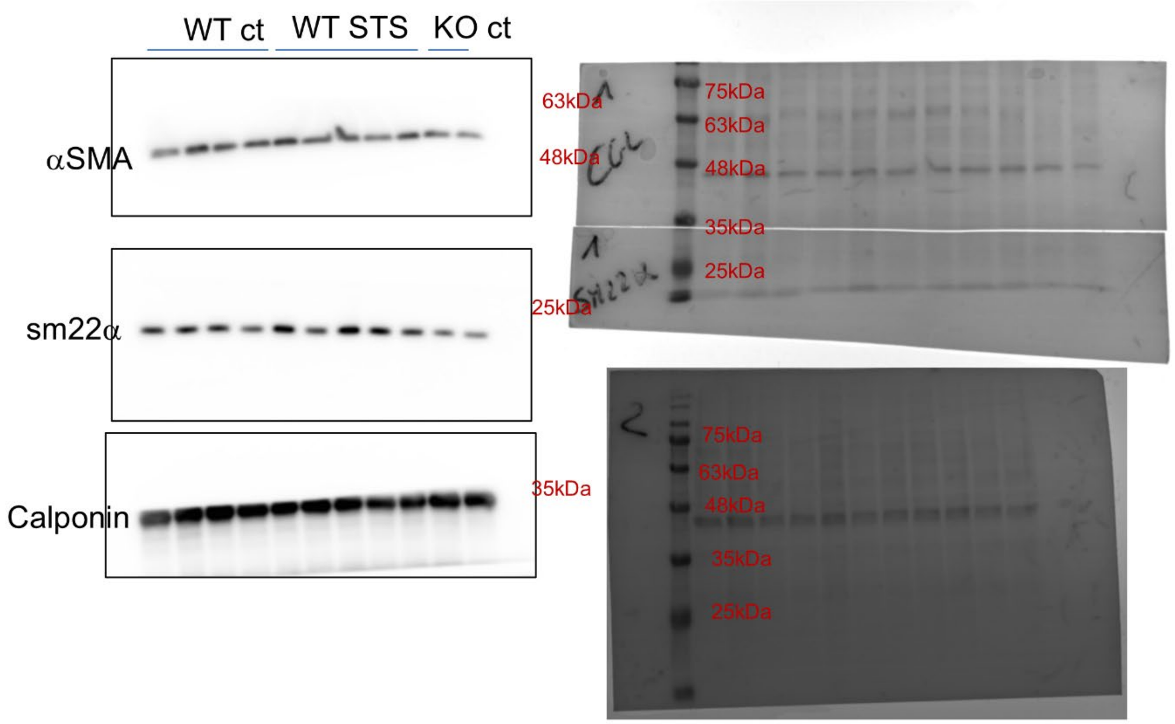

Supplementary Figure 8: original blots Supplementary Figure 7b

The blots were cut up prior to staining to allow for parallel staining of multiple proteins.

Multiple antibodies were successively blotted on the same blot

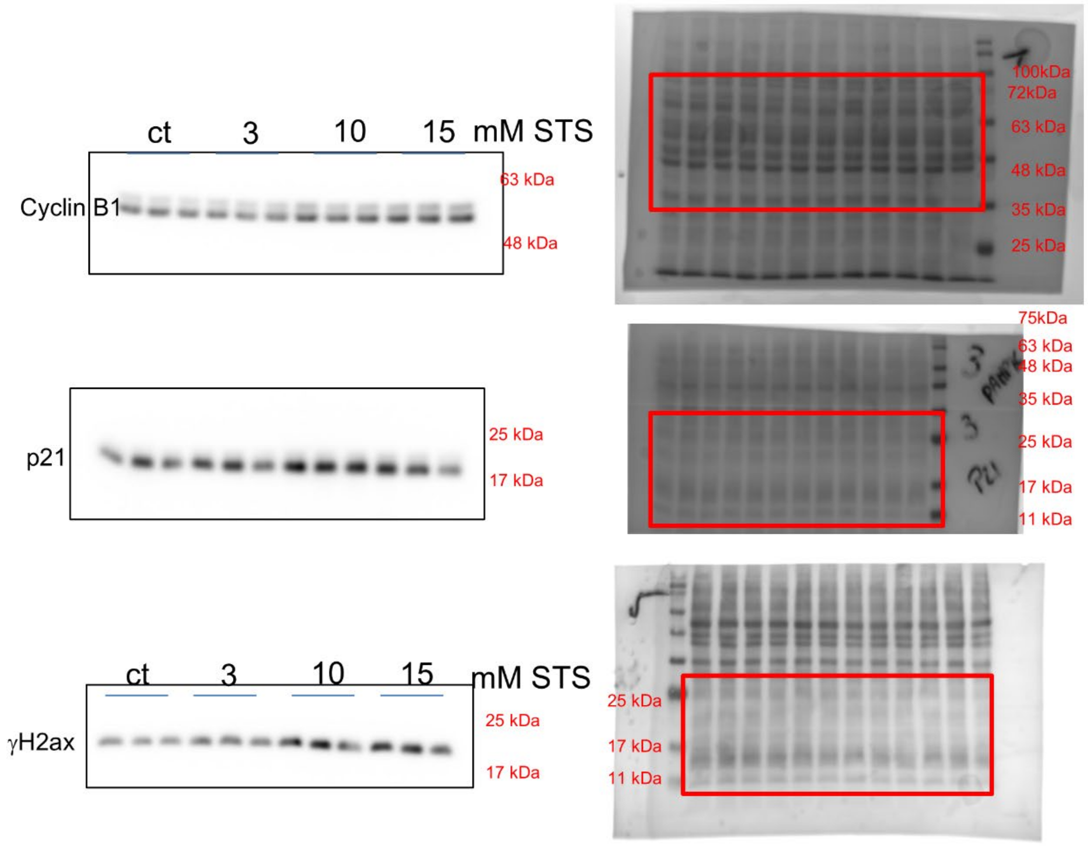

Supplementary Figure 9: original blots Figure 6b

The blots were cut up prior to staining to allow for parallel staining of multiple proteins.

Multiple antibodies were successively blotted on the same blot

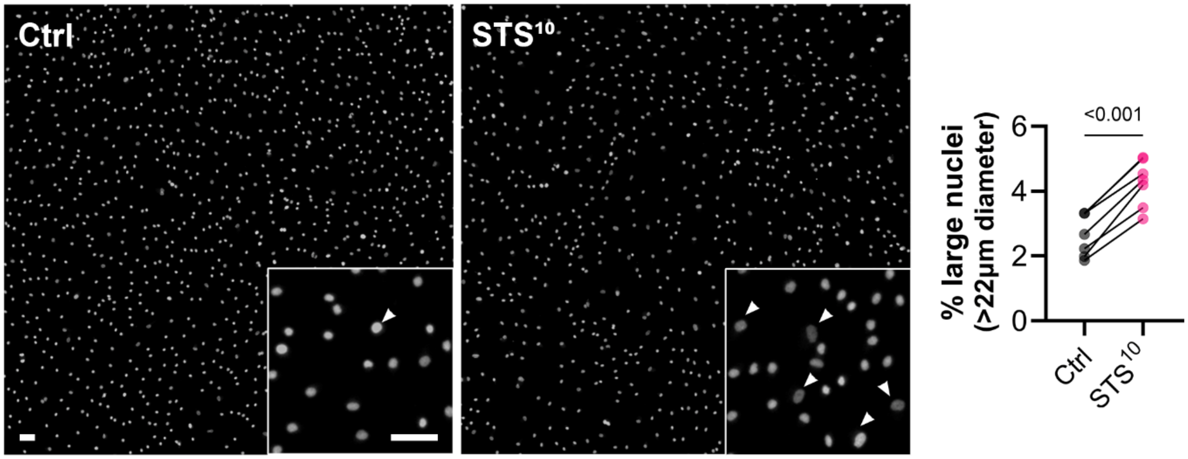

Supplementary Figure 10. STS promotes VSMC senescence

Left panel: representative live cell imaging of vascular smooth muscle cells (VSMC) nuclei (Hoescht staining) in cells exposed to 10mM sodium thiosulfates (STS^{10}) for 24 hours. Scale bar: 50 µm. *Right panel:* Data are % of large nuclei (diameter >22µm) in VSMC from 5 independent experiments. P value determined by bilateral paired t-test.

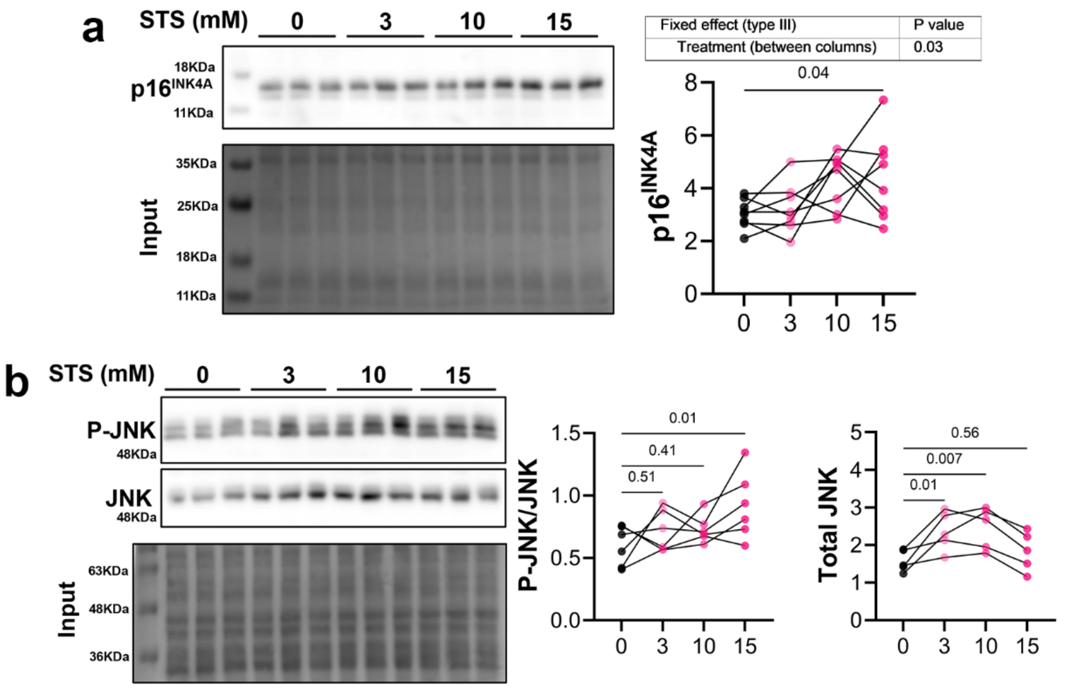

Supplementary Figure 11. STS promotes VSMC senescence

a-b) Western blot analysis of $P16^{INKA4}$, P-JNK and JNK in vascular smooth muscle cells (VSMC) treated for 24 hours with increasing concentration of sodium thiosulfate (STS), as indicated. P values determined by repeated measures REML followed by Dunnett's multiple comparisons tests from 8 (p16) or 5 (P-JNK and JNK) independent experiments.

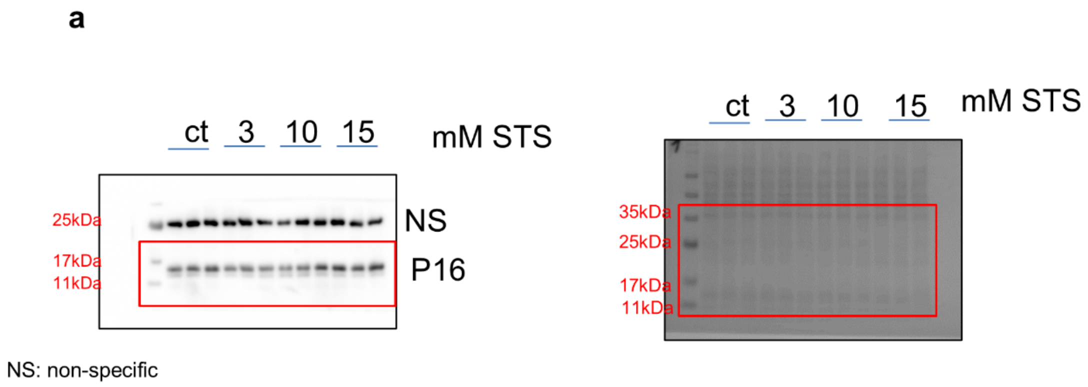

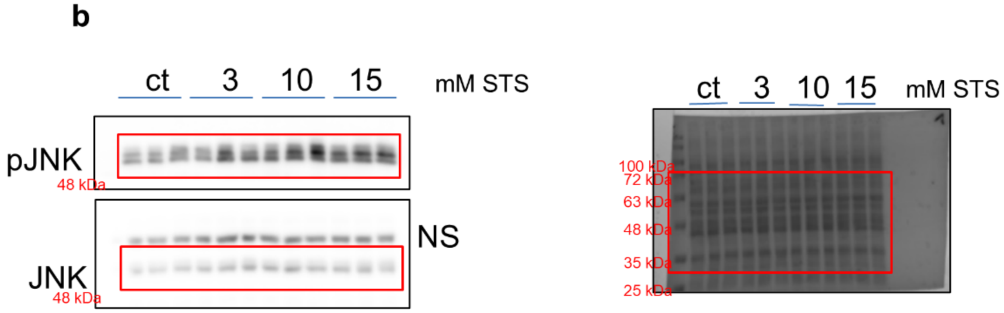

Supplementary Figure 12: original blots Supplementary Figure 11a and 11b

The blots were cut up prior to staining to allow for parallel staining of multiple proteins.

Multiple antibodies were successively blotted on the same blot.

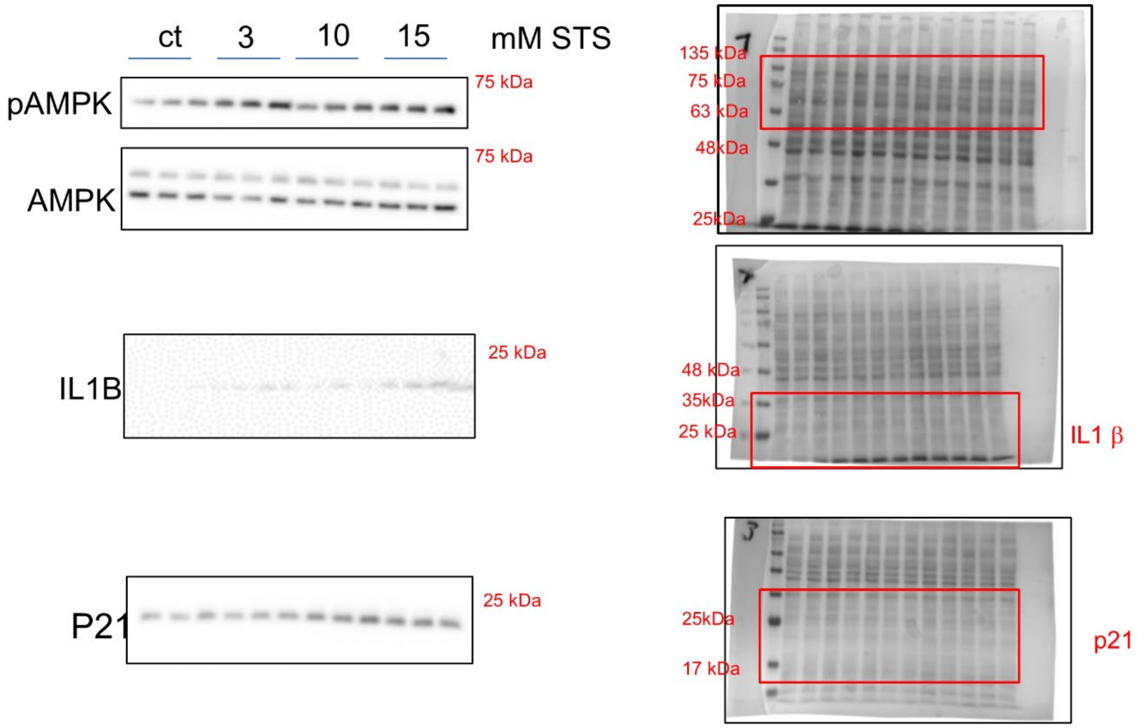

Supplementary Figure 13: original blots Supplementary Figure 6c

The blots were cut up prior to staining to allow for parallel staining of multiple proteins.

Multiple antibodies were successively blotted on the same blot

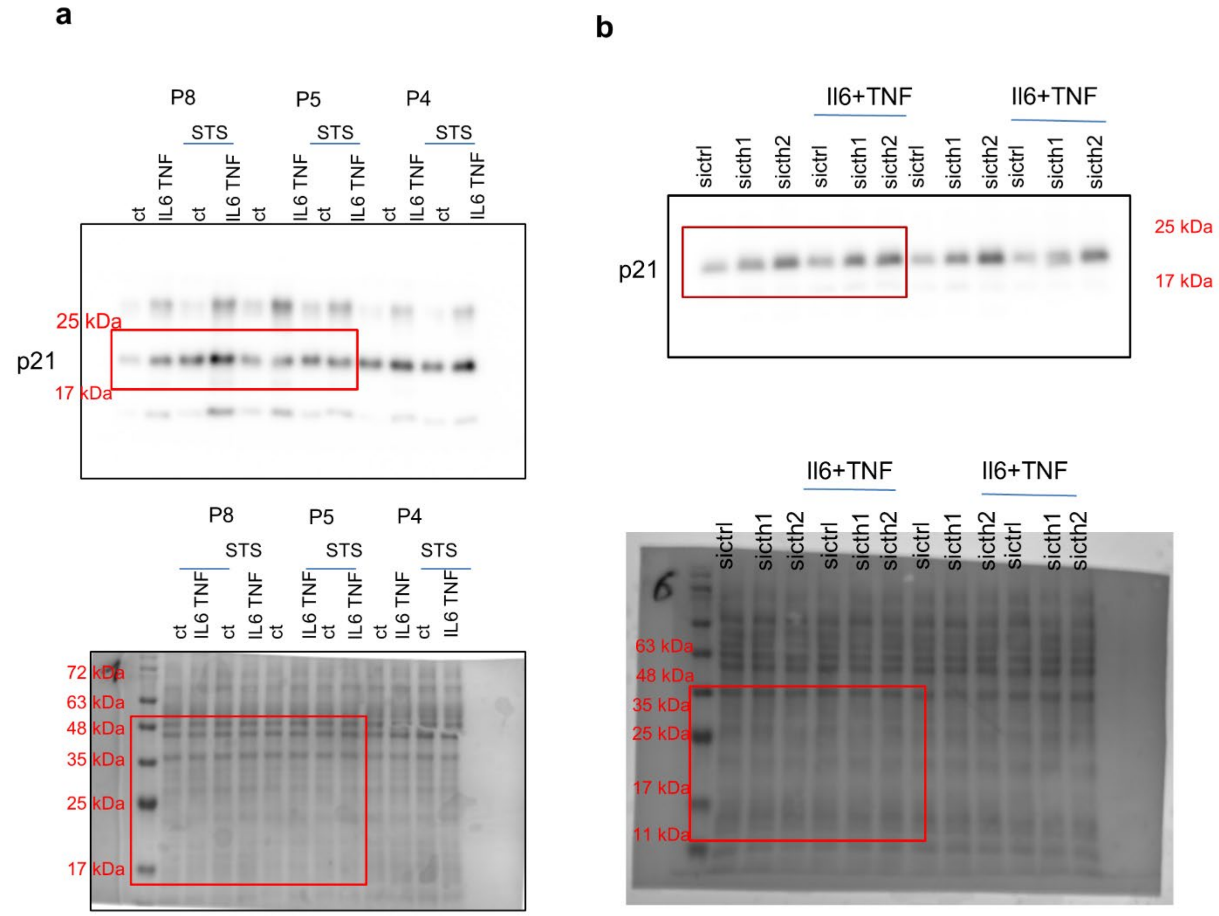

Supplementary Figure 14: original blots Supplementary Figure 7c and f

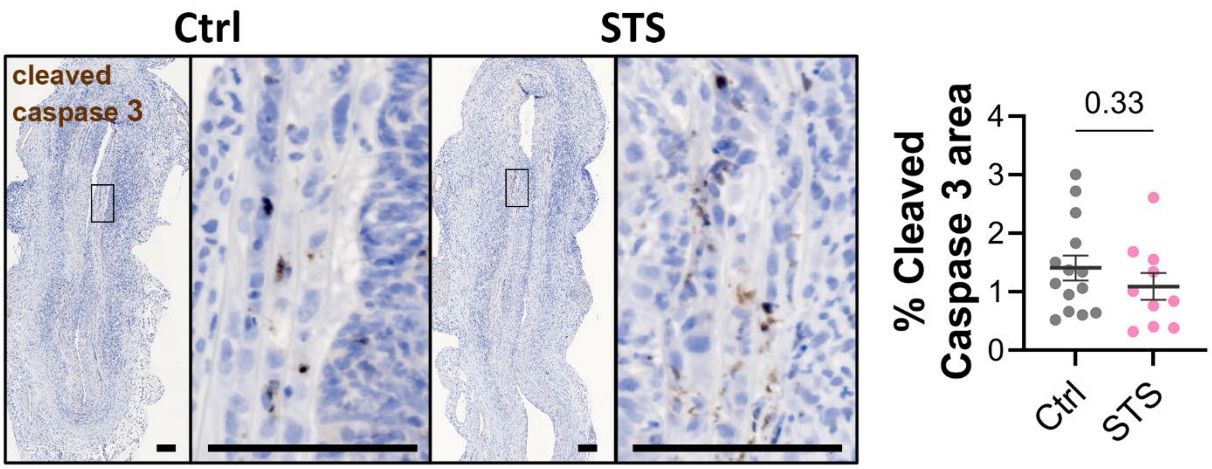

Supplementary Figure 15. Cleaved caspase 3 staining reveal little or no apoptosis in the aortic wall of AAA mice.

Left Panels: Representative Cleaved Caspase 3 immunostaining in sub-renal mouse AAA in wild type male mice with topical elastase application treated with sodium thiosulfate (STS) or not (Ctrl). Right insets are 5-fold magnifications of left images. Scale bar = 80 µm. *Right Panels*: quantitative assessment of Cleaved Caspase 3 immunostaining in 15 Ctrl and 10 STS-treated mice. Data are mean±SEM of. No significant difference as determined by bilateral unpaired t-test.

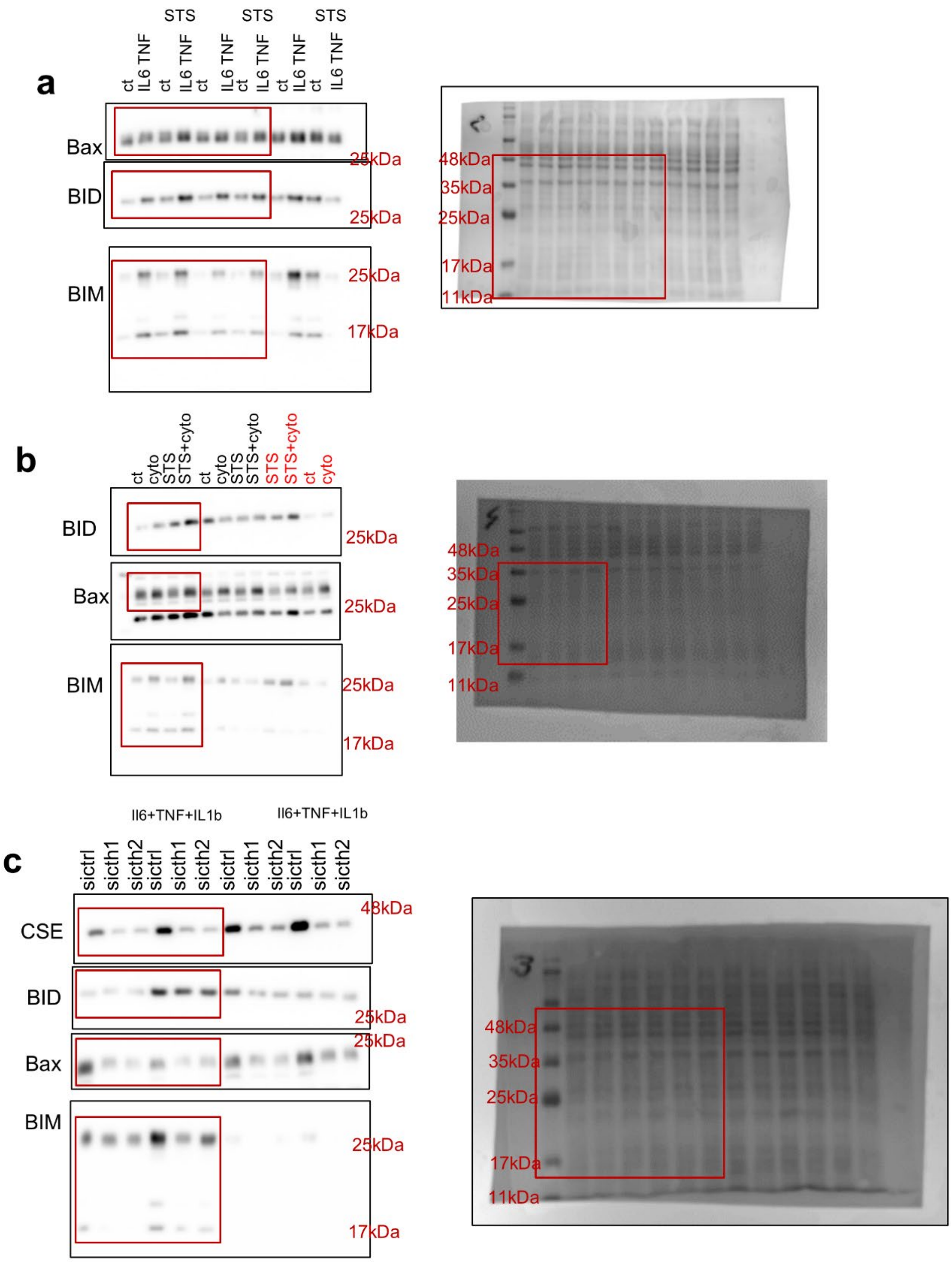

Supplementary Figure 16: original blots Figure 7c and f

The blots were cut up prior to staining to allow for parallel staining of multiple proteins.

Multiple antibodies were successively blotted on the same blot
