# Supplementary material for: Hydrogen sulfide aggravates neutrophil infiltration, vascular remodeling and elastase-induced abdominal aortic aneurysm in male mice
Source: Commun Med (Lond). 2025 Jul 1;5:267. doi: 10.1038/s43856-025-00978-5 (PMC12217252; doi:10.1038/s43856-025-00978-5)
Supplement: Supplementary file 3 — Description of Additional Supplementary Files [file 43856_2025_978_MOESM3_ESM.pdf]

Description of Additional Supplementary Files

File name- Supplementary Data 1

File description- Raw numerical data (source data) are available in Supplementary Data 1.

File name- Supplementary Data 2

File description- A list of models, reagents, chemicals, and equipment is included in the Supplementary Data 2

File name- Supplementary Data 3

File description- The antibodies used in that study are described in Supplementary Data 3
